# Evaluating pro-arrhythmogenic effects of the T634S-hERG mutation: insights from a simulation study

**DOI:** 10.1098/rsfs.2023.0035

**Published:** 2023-12-15

**Authors:** Wei Hu, Wenfeng Zhang, Kevin Zhang, Ehab Al-Moubarak, Yihong Zhang, Stephen C. Harmer, Jules C. Hancox, Henggui Zhang

**Affiliations:** ^1^ Biological Physics Group, Department of Physics and Astronomy, University of Manchester, Manchester M13 9PL, UK; ^2^ College of Computer and Information Science, Chongqing Normal University, Chongqing, People's Republic of China; ^3^ Southmead Hospital, North Bristol Trust, Bristol, UK; ^4^ School of Physiology, Pharmacology and Neuroscience, University of Bristol, Biomedical Sciences Building, University Walk, Bristol BS8 1TD, UK; ^5^ Key Laboratory of Medical Electrophysiology of Ministry of Education and Medical Electrophysiological Key Laboratory of Sichuan Province, Institute of Cardiovascular Research, Southwest Medical University, Luzhou 646000, People's Republic of China; ^6^ Beijing Academy of Artificial Intelligence, Beijing 100084, People's Republic of China

**Keywords:** hERG, KCNH2, long QT syndrome, human ventricles, ventricular fibrillation, cardiac modelling

## Abstract

A mutation to serine of a conserved threonine (T634S) in the hERG K^+^ channel S6 pore region has been identified as a variant of uncertain significance, showing a loss-of-function effect. However, its potential consequences for ventricular excitation and arrhythmogenesis have not been reported. This study evaluated possible functional effects of the T634S-hERG mutation on ventricular excitation and arrhythmogenesis by using multi-scale computer models of the human ventricle. A Markov chain model of the rapid delayed rectifier potassium current (I_Kr_) was reconstructed for wild-type and T634S-hERG mutant conditions and incorporated into the ten Tusscher *et al*. models of human ventricles at cell and tissue (1D, 2D and 3D) levels. Possible functional impacts of the T634S-hERG mutation were evaluated by its effects on action potential durations (APDs) and their rate-dependence (APDr) at the cell level; and on the QT interval of pseudo-ECGs, tissue vulnerability to unidirectional conduction block (VW), spiral wave dynamics and repolarization dispersion at the tissue level. It was found that the T634S-hERG mutation prolonged cellular APDs, steepened APDr, prolonged the QT interval, increased VW, destablized re-entry and augmented repolarization dispersion across the ventricle. Collectively, these results imply potential pro-arrhythmic effects of the T634S-hERG mutation, consistent with LQT2.

**Key Points**

— An updated Markov chain model of wild-type I_Kr_ and a model of variant of uncertain significance (T634S-hERG) have been developed and validated.
— Reduced I_Kr_ due to the T634S-hERG mutation prolonged the action potential duration (APD) of human ventricular cells. The mutation also steepened the rate-dependence curve of APD, which facilitates the genesis of APD alternans.
— At the tissue level, the mutation prolonged the QT interval of simulated pseudo-ECG, increased tissue vulnerability to unidirectional conduction block in response to a premature stimulus, indicating an increased tissue susceptibility to the genesis of re-entrant excitation waves. It also destabilized re-entry leading to multiple wavelets that underlie ventricular arrhythmia.
— In the three-dimensional model of the human ventricles, the T634S-hERG mutation produced increased dispersion of ventricular repolarization, which is pro-arrhythmic.

## 1. Introduction

Long QT syndrome (LQTS) is a potentially lethal cardiac repolarization disorder linked causally to sudden cardiac death, particularly in young people [1]. Previous studies have shown that LQTS is more prevalent in patients with autoimmune deficiency disorders, including systemic lupus erythematosus and Sjogren's syndrome [2–4]. In practice, LQTS manifests as a prolonged QT interval (i.e. the time interval between the beginning of the QRS complex and the end of T-wave) on the electrocardiogram (ECG). A prolonged QT interval reflects impaired cardiac repolarization that may increase tissue susceptibility to arrhythmogenesis and sudden cardiac death. Acquired LQTS is typically attributable to medications that inhibit the activity of potassium channels encoded by *human Ether-à-go-go Related Gene* (*hERG*) [5–7]. Congenital LQTS is linked to mutations of proteins that influence cardiac electrogenesis. So far, 17 types of LQTS have been identified [8], although a recent re-evaluation has suggested that only three genes (*KCNQ1*,*KCNH2*, *SCN5A*) have definitive evidence of causality of typical LQTS [9].

The most frequently observed forms of congenital LQTS arise from mutations to *KCNQ1* and *hERG* (alternative nomenclature *KCNH2*), which respectively contribute to the slow delayed rectifier potassium current (I_Ks_, LQT1) and the rapid delayed rectifier potassium current (I_Kr_, LQT2); these account for 44% and 35% of cases, respectively [9]. As the *hERG* gene encodes the cardiac potassium channel K_V11.1_ that mediates I_Kr_ [10–12], it plays a crucial role in cardiac repolarization [13,14]. Loss- and gain-of-function *hERG* mutations, respectively, underlie LQT2 [15,16] and the SQT1 variant of short QT syndrome [15,17].

LQT1 and LQT2 have distinct clinical cardiac phenotypes. LQT1 is usually triggered during exercise and rarely by rest/sleep, whereas LQT2 is rarely triggered by exercise and usually occurs in response to emotional stress [18]. Moreover, β-blocker therapy is effective in treating both subgroups [19], but the effect is more pronounced in LQT1 than in LQT2 patients [20,21].

Numerous *hERG* mutations have been identified in patients with LQT2 [22–25]. The reduction in functional I_Kr_ in LQT2 is associated with an increased risk of fatal arrhythmias [17,26–29]. However, while over 1000 hERG variants have been reported on public databases, for only a small fraction of them is their impact on I_Kr_ channel or ventricular repolarization known; the functional role for many variants remains unelucidated. A missense variant T634A to a conserved threonine residue in the hERG channel pore has been reported in an adolescent LQTS patient [30]. Recently, a T634S variant of uncertain significance (VUS) at the same residue was reported [30]. Patch-clamp characterization of T634S-hERG in HEK 293 cells found that this mutation, both alone and in combination with wild-type (WT) hERG, decreased hERG current (I_hERG_) [30]. This in turn led to prolonged action potential duration (APD) in a ventricular cell AP model [30]. However, simulations were not conducted to determine pro-arrhythmic effects of the mutation and at present any such effects remain unclear. Consequently, the aim of this study was to evaluate effects of the T634S-hERG mutation on ventricular repolarization and arrhythmogenesis, through use of multi-scale computational models of human ventricles.

## 2. Methods

Multi-scale mathematical models of the heart provide an alternative tool to investigate potential functional impacts of gene mutations on cardiac electrical excitation, electrical wave conduction and arrhythmogenesis when phenotypically accurate animal models are lacking. Such multi-scale cardiac models have been previously demonstrated to be beneficial in identifying molecular mechanisms of channelopathies (e.g. [31–35]).

### 2.1. Markov chain model of I_Kr_

We first developed a set of Markov chain (MC) models for describing I_Kr_ channel activation/inactivation kinetics in WT and *KCNH2* T634S mutation conditions by updating previous MC I_Kr_ formulations [31,32]. The states, state transitions and their associated transition rates of the MC I_Kr_ model are described in electronic supplementary material, figure S1, which comprise three closed states (C1, C2 and C3), one inactive state (I) and one open state (O). Based on experimental data [30], a set of equations and parameters for the state transition rates of the MC I_Kr_ model were obtained for WT and heterozygous (WT-T634S) mutation conditions (see electronic supplementary material, tables S1 and S2) by minimizing the least-squared difference between the experimental and simulation data of I_Kr_ time traces during voltage-clamp, using the Broyden–Fletcher–Goldfarb–Shanno (BFGS) algorithm [36]. As the experimental data [30] showed that in homozygous T634S mutation condition, the measured current was tiny, in simulations, it was simulated by fully eliminating I_Kr_. To validate the developed MC I_Kr_ models, the current–voltage (I–V) relationship curves were simulated by using the same voltage-clamp protocol as used experimentally and were compared to experimental data of [30] for WT and T634S-hERG, respectively (see electronic supplementary material, figures S2 and S3).

### 2.2. Single-cell model

#### 2.2.1. Single-cell model

The ten Tusscher human ventricular (TNNP) model [33] was used as a basal model, which was modified to incorporate the MC I_Kr_ model for WT and T634S-hERG conditions. The TNNP model was chosen as it was based on experimental data from human ventricular cells (where data were available) and validated by its ability to simulate human ventricular action potentials of epicardial (EPI), mid-myocardial (MIDDLE or MCELL) and endocardial (ENDO) cells. Moreover, the TNNP model has also been demonstrated previously to be suitable to simulate the functional effects of hERG mutations, such as the pro-arrhythmic effects of *KCNH2* N588K mutation [31]. Over the years, the TNNP model has been updated [31,32] and implemented to simulate electrical excitation waves at both tissue and organ levels as well [34,35].

In this study, we followed previous studies [34,37] to consider three distinct cell types in the transmural ventricular myocardium, which consists of EPI, MIDDLE and ENDO regions [34,37–39], and considered inhomogeneous I_Kr_ densities among them, with a ratio of G_Kr_ among the three regions as ENDO : MIDDLE : EPI = 1.0 : 0.8 : 1.3 [40].

#### 2.2.2. Stimulation protocol

An S1–S2 stimulation protocol was employed to evoke action potentials, from which the effect of the T634S-hERG mutation on the duration of action potential at 90% repolarization (APD_90_) was computed. Specifically, a series of conditioning S1 stimuli with a basic cycle length (BCL) of 1 s were first applied to excite the cell model until its solutions reached a steady state. Later, after an appropriate diastolic interval (DI), the S2 stimulus (with the same stimulation amplitude as the S1s) was applied to the cell model. The APD_90_ from the S2-evoked AP was computed in WT, homozygous and heterozygous T634S-hERG conditions with different DIs. When the DI was sufficiently small and fell into the refractory period of the S1-evoked AP repolarization, two S2-stimuli were implemented, which resulted in alternating action potentials, with one having a greater APD than the other. Therefore, at a fixed DI, two APD values were registered, producing a bifurcation of the APD restitution curve. Such bifurcation in the APD restitution curve has been observed experimentally and numerically [41,42]. In simulations, time-courses of APs, I_Kr_ and other major ion channel currents underlying APs were also recorded.

### 2.3. Tissue models

The reaction–diffusion equation was used to simulate excitation wave conduction in ventricular tissues as shown in equation (2.1) [31,43]:

2.1 $$\frac{\partial V_{m}}{\partial t}=\nabla \cdot (D\nabla V_{m})-\frac{I_{\mathrm{ion}}}{C_{m}},$$

where $V_{m}$ is the transmembrane potential, $I_{\mathrm{ion}}$ the total membrane ion channel current and $C_{m}$ the membrane capacitance. In the equation, ∇  is the spatial gradient operator and *D* is the electrical diffusion tensor that simulate the intercellular electrical coupling. Using equation (2.1), one-dimensional (1D), two-dimensional (2D) and three-dimensional (3D) tissue models were constructed as described below.

#### 2.3.1. One-dimensional model

A one-dimensional model of a transmural ventricular strand with a length of 21 mm was developed. The strand was discretized by a spatial resolution of 0.15 mm forming 140 nodes, each of which was simulated by the TNNP model. The 1D strand model considered the transmural electrical heterogeneity by implementing three distinctive cell regions, including ENDO, MIDDLE and EPI cells with a proportion ratio of 1 : 1.3 : 1 as used in our previous studies [31,32]. Using the 1D tissue model, effects of the T634S-hERG mutation on the QT interval of simulated pseudo-ECGs, tissue vulnerable window (VW, a measure of inducibility of re-entrant excitation) were evaluated using the same method as used in previous studies [44–46] and details are presented in the electronic supplementary material, §§4 and 5.

#### 2.3.2. Two-dimensional tissue model

The 2D model considered an idealized inhomogeneous ventricular tissue slice with a size of a 7.5 × 7.5 cm^2^, which was also discretized by a spatial resolution of 0.15 mm forming a grid of 500 × 500 nodes, each of which was simulated by the TNNP model of ventricular cells. In this model, isotropic tissue conduction property was considered; therefore a scalar value of *D* was used and set to 0.18 mm^2^ ms^−1^ producing a conduction velocity of 0.7 m s^−1^ as used in our previous studies [31,47]. The 2D tissue model was composed of ENDO, MIDDLE and EPI cell regions with the same proportion ratio as in the 1D strand model. The 2D model of the reaction–diffusion equation (equation (2.1)) was numerically solved by a finite difference method with a time step of 0.02 ms which produced a stable solution as shown in our previous studies [31,43]. In simulations, the 2D model was used to investigate the functional effects of the T634S-hERG mutation on the dynamic behaviours of spiral waves, such as the stability and stationarity, frequency spectrum and meandering patterns of spiral waves. Details for tracing tip trajectory of spiral waves for repolarization the meandering patterns of spiral waves are presented in the electronic supplementary material, §5.

#### 2.3.3. Stimulus protocol for initiation of spiral waves

Spiral waves in the 2D tissue model were initiated by a standard S1–S2 stimulation protocol. To begin with, a series of S1 stimuli were employed on the left side of the tissue slice to induce a planar wave. Then, after a time interval, an S2 stimulus was applied to the top half of the tissue model with a cross direction to the S1. This simulation setup allowed for controlled initiation of re-entrant waves. The S1-evoked planar wavefront was broken by the S2-evoked excitation wave, with the broken ends forming the cores of a spiral wave.

#### 2.3.4. Three-dimensional ventricle model

The 3D model of the human ventricle used in this study was developed by incorporating the single-cell model of the human ventricle developed by ten Tusscher *et al*. [34,35,48] into a 3D realistic ventricular geometry constructed by triple down-sampling DT-MRI scans of the human ventricles [49]. The 3D geometry was discretized by a spatial resolution of 0.2 mm forming 7.2 million nodes, each of which was simulated using the TNNP cell model.

To consider cellular electrical heterogeneity of ventricular cells in the 3D anatomical model, transmural ventricular tissue was segmented into three different regions: EPI, MIDDLE and ENDO cell regions, using a proportional ratio of 1 : 1.3 : 1 as used in our previous studies [50]. In the 3D model, a pace-stimulation method was used to evoke excitation waves and the phase-distribution method [51,52] was used to initiate re-entrant excitation waves (see electronic supplementary material, figure S5, and other details in the electronic supplementary material, §7).

#### 2.3.5. Validation of the model at multi-scale levels

Details of validation of the developed models at the cellular, tissue and 3D organ levels are provided in the electronic supplementary material, table S3 in §8.

## 3. Results

**Figure 1.**
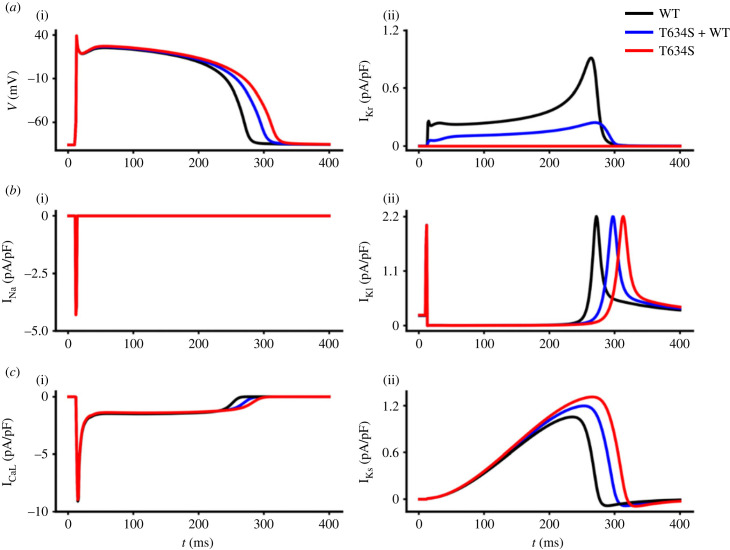
Simulated ventricular action potentials and major underlying ion channel currents in WT (black, 274.1 ms for APD_90_), heterozygous (T634S + WT; blue, 301.8 ms for APD_90_) and homozygous (T634S; red, 316.6 ms for APD_90_) mutation conditions with a pacing frequency of 1 Hz. *a*(i) Time traces of APs at steady state. *a*(ii) I_Kr_, *b*(i) I_Na_, *b*(ii) I_K1_, *c*(i) I_CaL_ and *c*(ii) I_Ks_.

Figure 1 presents the time courses of ventricular action potentials computed from the TNNP EPI cell model in WT (black), heterozygous (T634S + WT; blue) and homozygous (I_Kr_ was set to 0; red) mutation conditions, and some of the corresponding ion channel currents. It was shown that the reduced I_Kr_ due to the T634S-hERG mutation slowed ventricular AP repolarization, leading to a prolonged APD for both heterozygous and homozygous mutation conditions (figure 1*a*(i)) compared to the WT case. In the case of heterozygous mutation, the measured APD_90_ was prolonged by 10.1% in EPI cells, 13.6% in MIDDLE cells and 10.3% in ENDO cells. In the homozygous mutation case, the measured APD_90_ was prolonged by 15.5% in EPI, 31.3% in MIDDLE and 17.9% in ENDO cells, respectively. Such an APD prolongation by the T634S-hERG mutation was broadly similar to the observation of Al-Moubarak *et al*. using a simple approach of reduced I_Kr_ conductance (g_Kr_) [30] (figure 1*a*(ii)); the amplitude of I_Kr_ during the AP time course was reduced by approximately 64% in the heterozygous T634S + WT mutation relative to WT.

The observed APD prolongation was also associated with secondary changes in some major ion channel currents underlying the AP. While there was no noticeable change in the maximal amplitude of I_Na_ (figure 1*b*(i)) or I_K1_ (figure 1*b*(ii)), an increase in the amplitude of I_CaL_ (figure 1*c*(i)) and I_Ks_ (figure 1*c*(ii)) was observed. The extent of observed APD prolongation was different between ENDO, MIDDLE and EPI cells, which altered the intrinsic APD differences among them (electronic supplementary material, figure S6). Such an inhomogeneous prolongation of ventricular APD may augment the dispersion of ventricular repolarization, leading to increased tissue susceptibility to re-entry, which was investigated in subsequent tissue simulations (below).

**Figure 2.**
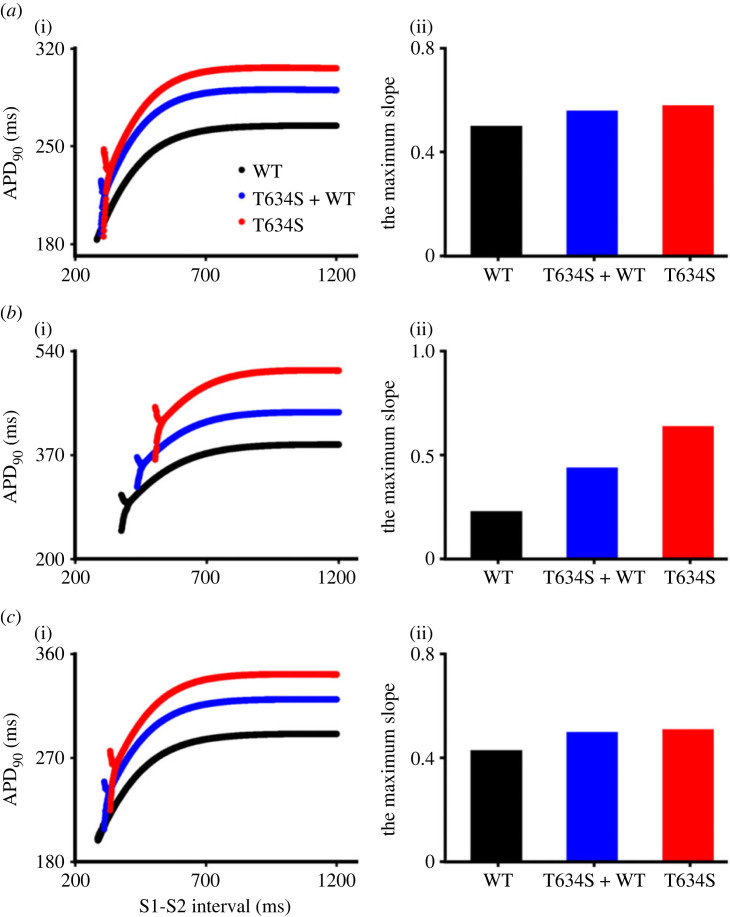
Computed APD restitution curves in the WT (black dotted line), heterozygous (T634S + WT; blue dotted line), and homozygous (T634S; red dotted line) mutation conditions from three different ventricular cell models. (*a*) EPI cell, (*b*) MIDDLE cell and (*c*) ENDO cell.

Figure 2 shows the effects of the T634S-hERG mutation on the rate dependence of APD (i.e. APD restitution; APDr) of ENDO (figure 2*a*(i)), MIDDLE (figure 2*b*(i)) and EPI (figure 2*c*(i)) cells. It was shown that across the whole range of SIs (i.e. the time interval between S1 and S2 stimuli) considered, the measured APD of all three cell models in heterozygous and homozygous mutation conditions were greater than that in WT condition. When the SI was reduced to below a threshold value, the computed APDr curve bifurcated, indicating the genesis of APD alternans, which was more pronounced in the mutation conditions than in the WT case. Note that the mutation had inhomogeneous effects on the three cell types, with greater impact on MIDDLE than the ENDO and EPI cell models, which was indicated by a right-ward shift of the threshold value of SIs at which the bifurcation occurred. The onset values of SI for the APDr curve of the three cell types to bifurcate in WT, heterozygous and homozygous T634S conditions are shown in electronic supplementary material, table S4, indicating an increased propensity for APD alternans to occur by the mutation as being manifested by occurrence of APD alternans at slower pacing rates (i.e. the greater SIs), implying pro-arrhythmic effects of the heterozygous and homozygous T634S-hERG conditions. Such an increased susceptibility of APD alternans in the T634S-hERG condition was attributable to the increased maximal slopes of the restitution curves as shown in figure 2*a*(ii)–*c*(ii) for ENDO, MIDDLE and EPI cells, respectively.

Using the 1D tissue model, we quantified the functional impacts of the T634S-hERG mutation on the simulated pseudo-ECG. It was shown that in both heterozygous and homozygous conditions, the T634S-hERG mutation increased the simulated QT interval, which changed from 354 ms in WT to 409 ms and 463 ms in heterozygous and homozygous mutation conditions (electronic supplementary material, figure S8), demonstrating a causal link between the reduction in I_Kr_ due to the T634S-hERG mutation and QT interval prolongation, which is a main feature of an LQTS phenotype.

**Figure 3.**
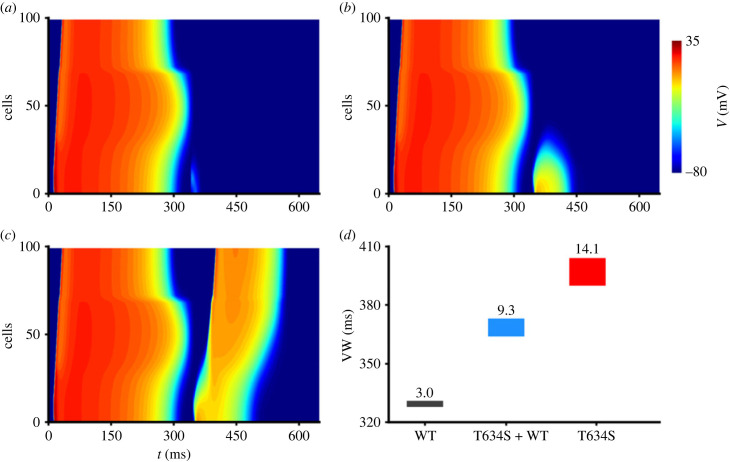
Effects of the T634S-hERG mutation on tissue vulnerability to arrhythmogenesis. (*a–c*) Space–time plot of AP propagation in a one-dimensional model of ventricular strand with S2 being applied to a local site of the strand (cell 4–14) after a time delay to the S1 stimulus that was applied to the ENDO end of the strand (cell 0). Depending on the time delay, the S2-evoked excitation wave was either bi-directionally blocked (*a*), or showed uni-directional conduction (*b*) or bi-directional conduction (*c*). (*d*) Measured width of the VW in WT (black), heterozygous (T634S + WT, blue) and homozygous (T634S, red) conditions.

Using the 1D model, we also quantified the functional impact of the T634S-hERG mutation on the inducibility of ventricular tissue to arrhythmogenesis by measuring the width of the vulnerable window (VW), during which a premature stimulus applied to the refractory tail of a preceding excitation wave evoked unidirectional conduction block. Results are shown in figure 3. Figure 3*a–c* shows the propagation of excitation waves in the 1D tissue model in response to an S2 stimulus being applied at different time delays after the S1 stimulus, which evoked an excitation wave propagating from the ENDO end of the tissue strand (cell 0) to the EPI end of the tissue (cell 100). With a time delay after the S1 stimulus, the S2 stimulus was applied to a local site of the strand (cell 4–14). When the time delay was short, the S2-evoked excitation failed to conduct as the tissue was in its refractory period (figure 3*a*). However, when the time delay was sufficiently large, the S2-evoked excitation wave propagated bi-directionally as the strand tissue had recovered its excitability from the preceding excitation (figure 3*c*). During a specific time window (i.e. VW), the S2-evoked excitation wave propagated in the retrograde direction but failed in the antegrade direction of the preceding excitation wave as the tissue only recovered its excitability in the former direction. Consequently, a uni-directional conduction (or block) was formed (figure 3*b*). The measured width of the VW (shown in figure 3*d*) was 3 ms in WT, which was increased to 9 ms in heterozygous and 14 ms in the homozygous mutation conditions.

Effects of the *KCNH2* T634S mutation on the dynamic behaviours of re-entrant excitation waves were further investigated in a 2D model of the human ventricular tissue. In simulations, the S1–S2 cross-stimulation protocol was used to initiate re-entrant spiral waves of excitation in both the WT and mutant tissue. Once initiated, the spiral waves were allowed to evolve for a period of 8 s, during which the characteristics of re-entrant excitation waves were quantified.

**Figure 4.**
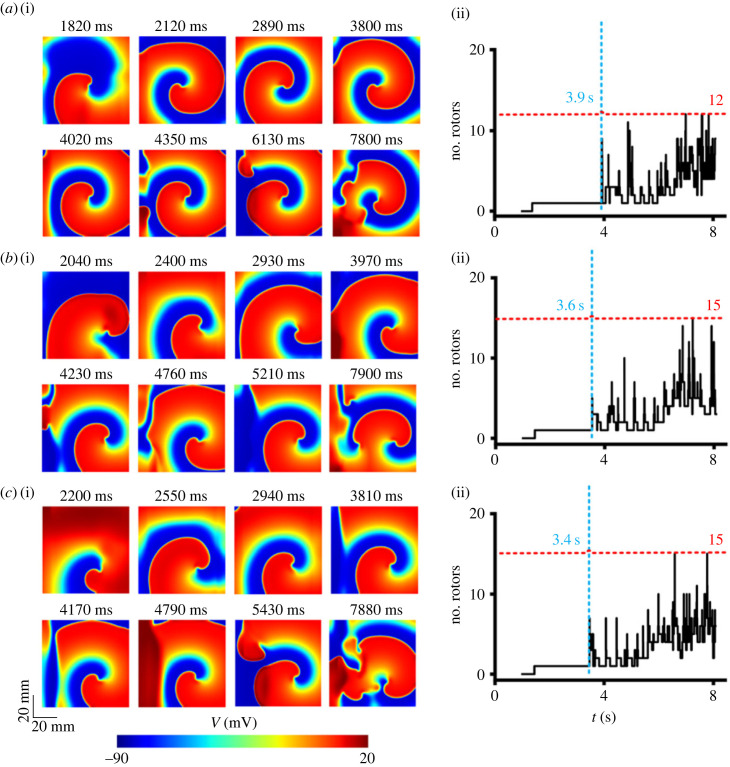
Snapshots of simulated re-entrant excitation waves in 2D ventricular tissue model, and the number of rotors (i.e. the number of tip cores of re-entry) in WT and mutant conditions. *a*(i) Snapshots in the WT condition. *b*(i) Snapshots in the heterozygous (T634S + WT) condition. *c*(i) Snapshots in the homozygous T634S condition. *a*(ii) Time trace of the number of spiral wave cores in the WT condition. *b*(ii) Time trace of the number of spiral wave cores in the heterozygous (T634S + WT) condition. *c*(ii) Time trace of the number of spiral wave cores in the homozygous T634S condition.

Figure 4 shows the snapshots of excitation waves in WT (figure 4*a*(i)), heterozygous (figure 4*b*(i)) and homozygous (figure 4*c*(i)) T634S-hERG mutation conditions. In all cases, the simulated spiral waves were unstable, leading to breakup of the re-entry forming multiple wavelets.

The time taken for the spiral wave to evolve into re-entrant wavelets was different among the WT, heterozygous and homozygous conditions. The T634S-hERG mutation accelerated the time course for the spiral wave to break down. In the WT case, the initiated spiral wave showed an initial period of meandering of 1820 ms. After that, there was a period of 2100 ms, during which the re-entry was relatively stable with smaller numbers of tips. At around 3900 ms, the spiral waves became progressively more unstable, forming multiple wavelets with multiple tips co-existing (figure 4*b*(i)).

In the mutant tissue, the initiated re-entrant waves were more unstable, which were reflected by the fact that the spiral wave started to break up at 3600 ms in the T634S heterozygous condition (figure 4*b*(ii)) and at 3400 ms in the homozygous condition (figure 4*b*(ii)–*c*(ii)), earlier than that of 3900 ms in WT condition. In the mutant tissue, re-entrant excitation waves were also more non-stationary, with a greater meandering area of tip trajectory in heterozygous and homozygous T634S-hERG mutation conditions (see electronic supplementary material, figure S9).

In the mutant tissue, it took a shorter period of time for multiple rotors to form and co-exist, suggesting that the T634S-hERG mutation effectively destablized the re-entrant excitation, leading to an increased maximum number of co-existing rotors (from 12 rotors in the WT case to 15 rotors in both mutant cases), suggesting an increased degree of un-coordinated ventricular excitation, a main feature of cardiac arrhythmias.

**Figure 5.**
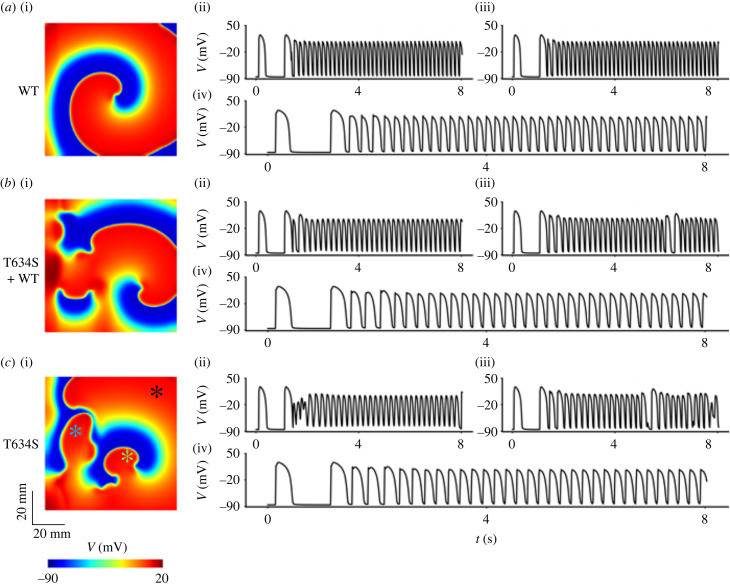
Time courses of APs recorded from local sites of ENDO, MIDDLE and EPI regions of the tissue model, which are marked with stars in panel *c*(i) for EPI (black), MIDDLE (green) and ENDO (blue). *a*(i) Snapshot of spiral wave in WT condition. *b*(i) Snapshot of spiral wave in heterozygous mutation (T634S + WT) condition. *c*(i) Snapshot of spiral wave in homozygous mutation (T634S) condition. *a*(ii)–(iv) Time courses of action potentials recorded from ENDO, MIDDLE and EPI regions of the tissue in WT condition. *b*(ii)–(iv) Time courses of action potentials recorded from ENDO, MIDDLE and EPI regions of the tissue in the heterozygous mutation (T634S + WT) condition. *c*(ii)–(iv) Time courses of action potentials recorded from ENDO, MIDDLE and EPI regions of the tissue in the homozygous mutation (T634S) condition.

Figure 5 shows the recorded time courses of APs at three different locations marked by the three stars in the 2D tissue model (figure 5*c*(i)), corresponding to regions of EPI (figure 5 for black star), MIDDLE (green star) and ENDO (blue star). In all regions, the AP time courses recorded in WT showed rapid but regular excitation over time (figure 5*a*(ii)), but the ones for heterozygous (figure 5*b*(ii)) and homozygous (figure 5*c*(ii)) mutation conditions were irregular, reflecting typical features of ventricular arrhythmias. Note that more irregular ventricular excitation was noticeable in the case of homozygous T634S-hERG mutation condition (figure 5*c*(ii)).

**Figure 6.**
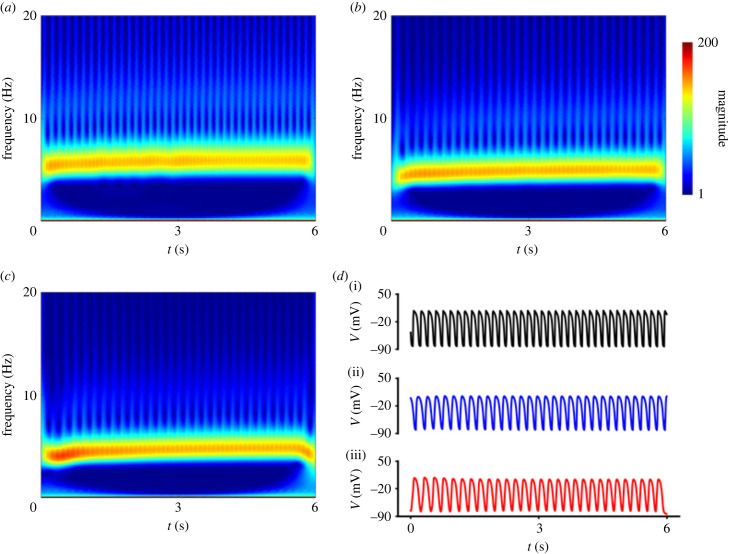
Time–frequency diagram of the AP time course recorded from the EPI region of the tissue model for WT and mutation conditions. (*a*) WT condition. (*b*) Heterozygous mutation (T634S + WT) condition. (*c*) Homozygous mutation (T634S) condition. *d*(i–iii) Corresponding AP time courses for WT (black), heterozygous (T634S + WT) (blue) and homozygous (T634S) (red) mutation conditions.

To investigate the impact of the T634S-hERG mutation on the excitation frequency, time–frequency analyses of the AP time courses recorded from the EPI region of the tissue were conducted. Results are shown in figure 6*a–c*. It was shown that the average frequency of re-entrant excitation waves was reduced by the mutation, which reduced from 5.5 Hz in the WT tissue to 4.8 Hz and 4.4 Hz in the heterozygous and homozygous mutant models, respectively, which are attributable to the prolonged APDs at the cellular level.

**Figure 7.**
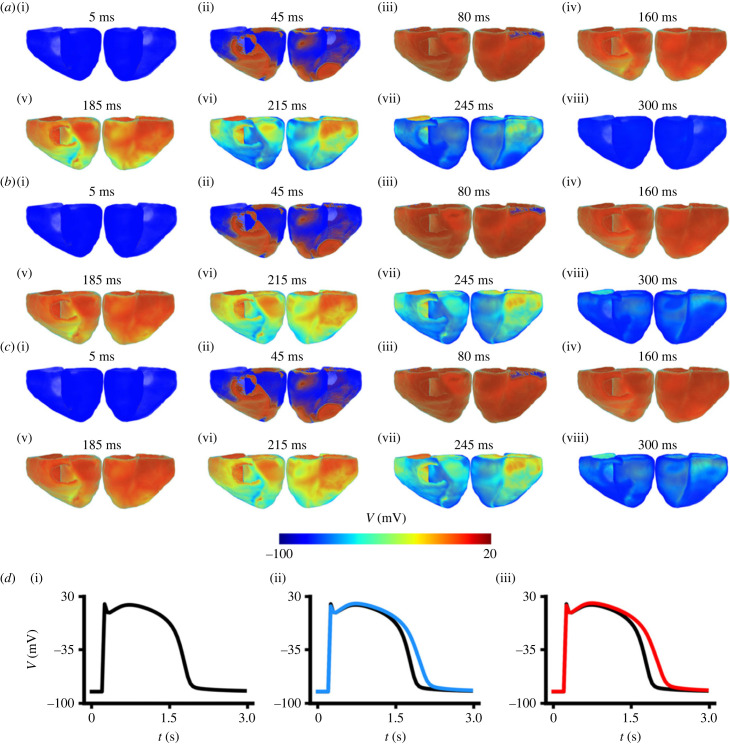
Snapshots of ventricular activation–repolarization in the WT and mutation conditions. (*a*) WT. (*b*) T634S + WT. (*c*) T634S condition. *d*(i–iii) Recordings of AP at the basal location of the left ventricle for WT (black), T634S + WT (blue) and T634S (red) conditions.

Using a 3D model of the human ventricles, potential effects of the T634S-hERG mutation on ventricular repolarization and repolarization patterns across the ventricular tissue were investigated. Figure 7 shows the snapshots of the propagation of ventricular excitations, which were evoked using the pacing-stimulation protocol described in the Methods section. For the WT case (figure 7*a*), the evoked excitation wave propagated following the same repolarization pattern as described in previous studies [31,53,54], starting first at the base of the anterior left ventricular wall, before then exciting the whole ventricles within a period of 90 ms (figure 7*a*(i)–(iii)).

**Figure 8.**
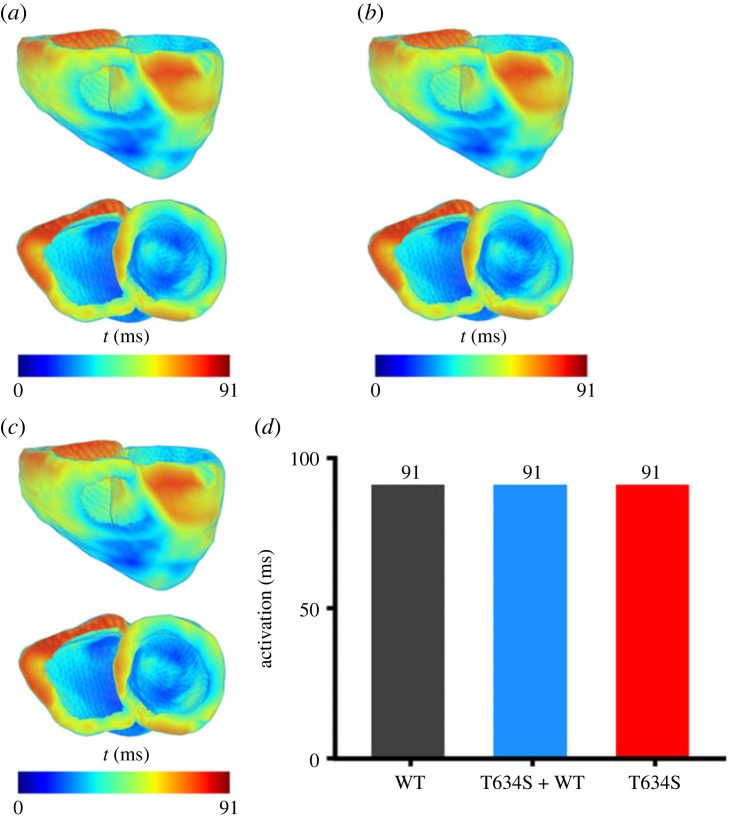
Colour coded timing distribution of activation across the ventricles in WT and mutation conditions. (*a*) WT. (*b*) Heterozygous (T634S + WT) condition. (*c*) Homozygous (T634S) condition. (*d*) Corresponding time interval taken for whole ventricles to be depolarized in WT (black), heterozygous (T634S + WT) (blue) and homozygous (T634S) (red) mutation conditions.

Noticeable repolarization started at around 190 ms (when activation of the ventricles completed) and completed at around 300 ms (figure 7*a*(vi)–(vii)) as shown in figure 7*a*(viii) in the WT condition. Both heterozygous and homozygous mutations delayed repolarization, but had no effect on the repolarization process as shown in figure 7*b*,*c*, respectively. The measured activation timing (i.e. the time taken for the whole ventricles to be fully excited) was about 91 ms for WT, heterozygous and homozygous mutations as shown in figure 8.

**Figure 9.**
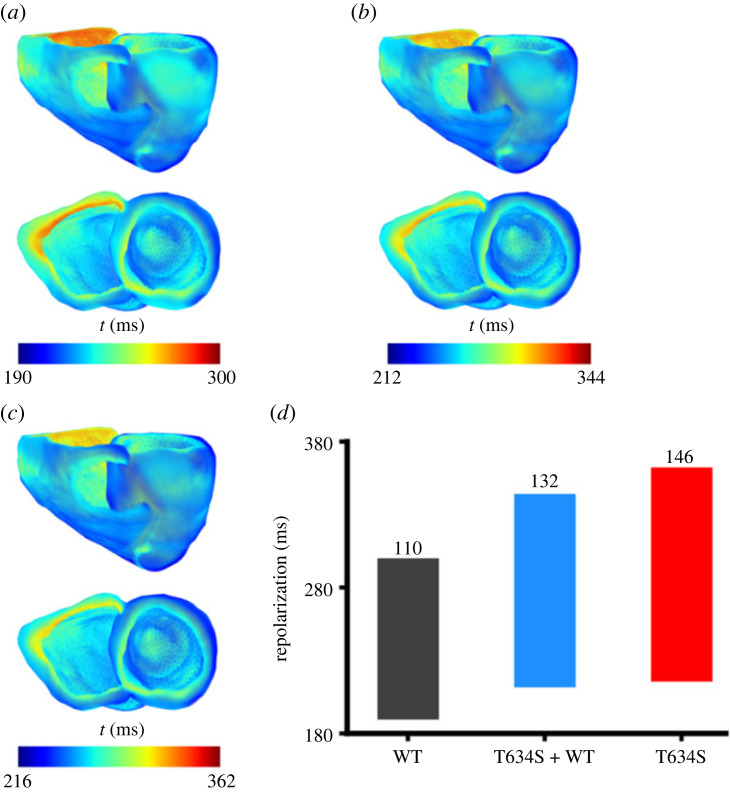
Colour coded timing distribution of ventricular repolarization in WT and mutation conditions. (*a*) WT. (*b*) Heterozygous (T634S + WT) mutation condition. (*c*) Homozygous (T634S) mutation condition. (*d*) Corresponding time interval for ventricles to becoming fully repolarized in WT (black), heterozygous (T634S + WT) (blue) and homozygous (T634S) (red) mutation conditions.

For the repolarization process, a greater dispersion in the recovery timing (i.e. the timing when tissue returned to resting potential) of the ventricles was observed under mutant conditions as shown in figure 9. In the WT case, the repolarization duration ranged from 190 to 300 ms (figure 9*a*), but changed to 212–344 ms in the heterozygous (figure 9*b*), and 216–362 ms in the homozygous (figure 9*c*) conditions. The repolarization interval also dramatically increased, changing from 110 ms in the WT condition to 132 ms in the heterozygous case to 146 ms in the homozygous condition (figure 9*d*).

**Figure 10.**
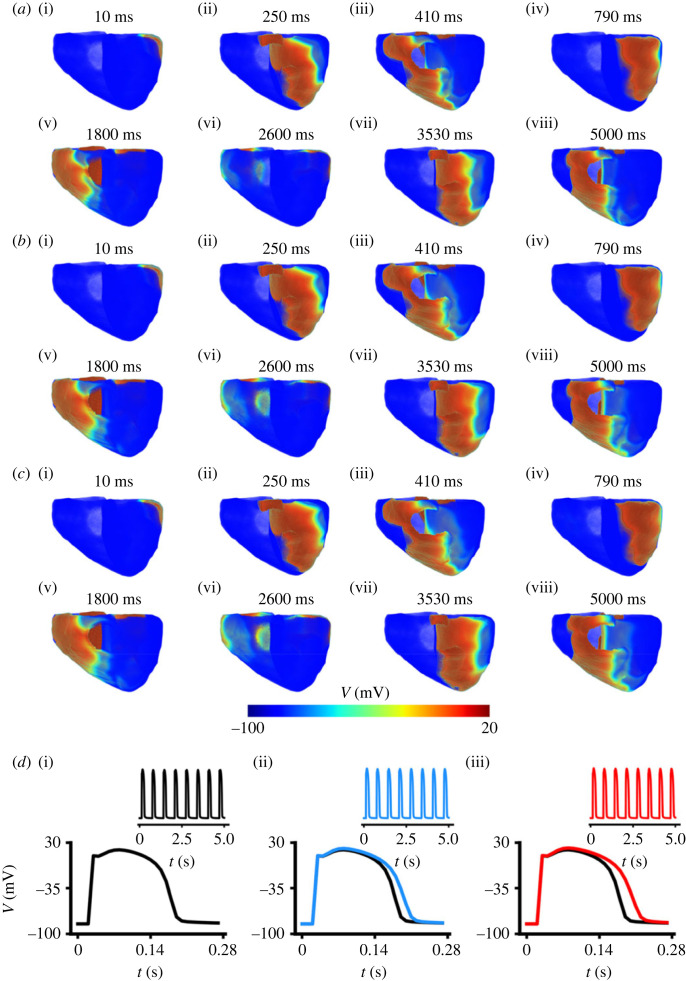
Snapshot of re-entry following initiation of scrolling waves in the 3D ventricular model in WT and mutation conditions. (*a*) WT. (*b*) Heterozygous (T634S + WT) mutation condition. (*c*) Homozygous (T634S) mutation condition. *d*(i)–(iii) APs recorded from a local site in the base of the left ventricle, and their corresponding time courses during 5 s for WT (black), heterozygous (T634S + WT) (blue) and homozygous (T634S) (red) mutation conditions.

As results from the 2D tissue model simulations of re-entry do not directly reflect those from the 3D anatomical model of the human ventricles, we further investigated potential impacts of the T634S-hERG mutation on re-entrant excitation waves in the 3D model with re-entrant excitation waves being initiated by using the phase mapping method as described in the Methods section. Results are shown in figure 10 for WT, heterozygous and homozygous mutation conditions. In all cases, the simulated re-entrant excitation waves were sustained for the whole period of 5 s simulation (figure 10*a*–*c*), but showed difference in the cycle length of rotation. The measured cycle length of the re-entrant excitation wave changed from 110 ms in WT to 132 ms and 146 ms in heterozygous and homozygous mutation conditions respectively, which was attributable to the lengthened APD as shown in figure 10*d*.

## 4. Discussion

### 4.1. Major findings

In this study, we implemented multi-scale computational models of the human ventricle to investigate potential impacts of the T634S-hERG mutation on ventricular electrical excitations and arrhythmogenesis at cellular and tissue levels. Our major contributions and findings are:

(1) The developed Markov chain model of I_Kr_ captured the functional impacts of T634S-hERG mutation on I_Kr_ channel properties.
(2) The loss-of-function effect on I_Kr_ of the T634S-hERG mutation prolonged ventricular AP repolarization, leading to an increased APD. It also altered ventricular APD restitution, leading to an increased maximal slope of the APDr curve and susceptibility to the genesis of AP alternans, which is pro-arrhythmic.
(3) The mutation prolonged the QT interval of simulated pseudo-ECGs, increased tissue vulnerability to initiation of uni-directional conduction block, and destabilized re-entrant excitation waves that led to more wavelets co-existing in ventricular tissue than that in the WT condition.
(4) The mutation augmented dispersion of ventricular repolarization across the ventricles that is pro-arrhythmic.

### 4.2. Mechanisms of pro-arrhythmogenesis of KCNH2 T634S mutation

The potential for the T634S-hERG mutation to favour ventricular arrhythmogenesis has been hypothesized [30], but not hitherto been directly demonstrated. However, previous computational studies have shown that reduced I_Kr_ arising from pharmacological inhibition [55,56], gene mutations [57,58], and those gene mutations in association with LQT2 particularly [59,60] on impaired ventricular excitation and contraction. In this study, our simulation results have shown that a reduced I_Kr_ due to the T634S-hERG mutation prolonged ventricular APD and QT interval, suggesting that the mutation may cause an LQT2 phenotype, and, therefore, is potentially arrhythmogenic [30,61,62]. Indeed, we have found from simulations that the mutation prolonged APD, altered the APD restitution properties of ventricular cells by increasing the maximal slope of the APDr, facilitating the genesis of AP alternans at fast pacing rates. Previous work has shown that conduction AP alternans in ventricular tissue can generate functional heterogeneity in tissue excitation that breaks down ventricular excitation waves, leading to the formation of re-entrant excitation waves underlying ventricular fibrillation [41,63].

Our simulation results have also shown that the T634S-hERG mutation increased the tissue vulnerable window for initiation of uni-directional conduction block in response to a premature stimulus, forming a substrate favouring the formation of re-entrant excitation. Such an increased VW may be attributable to an increased relative refractory period of ventricular excitation due to the prolonged APD. It has also been shown that the mutation had non-uniform impacts on modulating the APD among the ENDO, MIDDLE and EPI cells, with the biggest impact on the MIDDLE cell. Such a non-uniform APD prolongation among the three cell types augmented the transmural APD dispersion, which contributed to a pro-arrhythmic substrate in 3D simulations.

The pro-arrhythmic effects of the T634S-hERG mutation were also shown by their impact on the stability of re-entrant excitation waves. Simulation results from the 2D mutant tissue model showed that though the re-entry had relatively slower excitation rates due to prolonged repolarization of cellular action potentials, they broke down more easily, leading to a greater number of wavelets co-existing when compared with the WT condition. These co-existing multiple wavelets implied a higher degree of un-coordinated ventricular excitations that can manifest as ventricular fibrillation.

### 4.3. Relevance of the study

Malfunction of I_Kr_ alters cardiac repolarization. The simulation results from this study show that the reduced I_Kr_ arising from the T634S-hERG mutation prolonged ventricular cellular APD and the QT interval of the pseudo-ECG, augmented dispersion of ventricular repolarization, increased tissue inducibility to uni-directional conduction block and destabilized re-entrant excitation waves. Collectively these consequences underlie the pro-arrhythmic effects of the T634S-hERG mutation under both homozygous and heterozygous conditions, although in a clinical scenario patients are most likely to be heterozygous for such a mutation. These results indicate that such patients are likely to be at risk of ventricular arrhythmogenesis. Additionally, caution may be warranted for carriers of the mutation (particularly if they have prolonged QT intervals) to avoid drugs that are known to inhibit I_Kr_/hERG channels [64], due to potentially synergistic actions of the T634S-hERG mutation and I_Kr_/hERG channel blockers.

Recently, different approaches have been implemented to predict cellular phenotypes of genetic VUS that include the uses of machine learning [65–67] and high-throughput patch-clamp [68,69]. In future, such machine learning methods and data obtained by high-throughput patch-clamp can be used in conjunction with biophysically detailed multi-scale computer models of the heart to unveil the functional impact of genetic VUS on cardiac excitation and arrhythmogenesis.

### 4.4. Limitations of the study

There are some limitations in this study. The limitations of the ten Tusscher *et al*. model and its updates have been well documented elsewhere [33,48]. In this study, we developed a Markov I_Kr_ formulation for WT and T634S-hERG mutation conditions based on voltage-clamp data obtained from hERG channels heterologously expressed in a cell line [30], rather than from native human ventricular cells; therefore the validation of the MC I_Kr_ formulation was done by comparing the simulated relative change of I–V relationship to experimental data. Owing to a lack of available patient data, it was not possible to compare the simulated effects of the mutation on changed APD to QT intervals on a measured ECG. The implemented 2D tissue model was idealized, without considering the anisotropic conduction of cardiac tissue, which may influence the dynamic behaviour of re-entrant excitation waves. In addition, possible regional differences in cell membrane capacitance and electrical diffusion properties in the ventricular tissue were not considered in the monodomain equation. In the 3D model, we did not consider a 3D network structure incorporating Purkinje fibres, which may also play an important role for initiation and maintenance of re-entrant excitation waves. While it is necessary to make the limitations of the study explicit, nevertheless, they do not alter the core conclusions of the study.

## 5. Conclusion

In this study, we developed a Markov chain model of I_Kr_ channel for WT and T634S mutant hERG, based on experimental voltage clamp data. By incorporating the developed I_Kr_ formulation into multi-scale computational models of the human ventricles, we characterized the functional consequences of the mutation on ventricular excitation and arrhythmogenesis. Our results showed that the mutation, in both heterozygous and homozygous conditions, prolonged the AP repolarization process and QT interval of simulated pseudo-ECGs, increased tissue VW to re-entry and destabilized the re-entry forming multiple re-entrant wavelets. It also augmented ventricular repolarization dispersion which is also arrhythmogenic. In conclusion, our results provide insight into how this loss-of-function hERG mutation can both prolong ventricular repolarization and increase vulnerability to ventricular arrhythmia.

## Data Availability

Supplementary material is available online [70].

## References

[1] Schwartz PJ et al. 2009 Prevalence of the congenital long-QT syndrome. Circulation **120**, 1761-1767. (10.1161/CIRCULATIONAHA.109.863209) 19841298 PMC2784143

[2] Sgreccia A, Morelli S, Ferrante L, Perrone C, De Marzio P, De Vincentiis G, Scopinaro F. 1998 QT interval and QT dispersion in systemic sclerosis (scleroderma). J. Intern. Med. **243**, 127-132. 9566641

[3] Cardoso CR, Sales MA, Papi JA, Salles G. 2005 QT-interval parameters are increased in systemic lupus erythematosus patients. Lupus **14**, 846-852. (10.1191/0961203305lu2225oa) 16302681

[4] Szendrey J et al. 2019 Anti-Ro52 antibody acts on the S5-pore linker of hERG to chronically reduce channel expression. Cardiovasc. Res. **115**, 1500-1511. 30544220 10.1093/cvr/cvy310 PMC6648346

[5] De Waard S et al. 2020 Functional impact of BeKm-1, a high-affinity hERG blocker, on cardiomyocytes derived from human-induced pluripotent stem cells. Int. J. Mol. Sci. **21**, 7167. (10.3390/ijms21197167) 32998413 PMC7582727

[6] Hu W, Clark RB, Giles WR, Shibata E, Zhang H. 2021 Physiological roles of the rapidly activated delayed rectifier K^+^ current in adult mouse heart primary pacemaker activity. Int. J. Mol. Sci. **22**, 4761. (10.3390/ijms22094761) 33946248 PMC8124469

[7] Garg P et al. 2018 Genome editing of induced pluripotent stem cells to decipher cardiac channelopathy variant. J. Am. Coll. Cardiol. **72**, 62-75. (10.1016/j.jacc.2018.04.041) 29957233 PMC6050025

[8] Schwartz PJ, Crotti L, Insolia R. 2012 Long-QT syndrome: from genetics to management. Circul. Arrhyth. Electrophysiol. **5**, 868-877. (10.1161/CIRCEP.111.962019) PMC3461497 22895603

[9] Lemoine MD et al. 2018 Human induced pluripotent stem cell-derived engineered heart tissue as a sensitive test system for QT prolongation and arrhythmic triggers. Circul. Arrhyth. Electrophysiol. **11**, e006035. (10.1161/CIRCEP.117.006035) 29925535

[10] Sanguinetti MC, Jiang C, Curran ME, Keating MT. 1995 A mechanistic link between an inherited and an acquired cardiac arrhytmia: HERG encodes the I_Kr_ potassium channel. Cell **81**, 299-307. (10.1016/0092-8674(95)90340-2) 7736582

[11] van den Boogaard M et al. 2019 Identification and characterization of a transcribed distal enhancer involved in cardiac Kcnh2 regulation. Cell Rep. **28**, 2704-2714. (10.1016/j.celrep.2019.08.007) 31484079

[12] Kozek KA et al. 2020 High-throughput discovery of trafficking-deficient variants in the cardiac potassium channel KV11.1. Heart Rhythm **17**, 2180-2189. (10.1016/j.hrthm.2020.05.041) 32522694 PMC7704534

[13] Chapman H, Ramström C, Korhonen L, Laine M, Wann KT, Lindholm D, Pasternack M, Törnquist K. 2005 Downregulation of the HERG (KCNH2) K^+^ channel by ceramide: evidence for ubiquitin-mediated lysosomal degradation. J. Cell Sci. **118**, 5325-5334. (10.1242/jcs.02635) 16263765

[14] Wilde AA, Bezzina CR. 2005 Genetics of cardiac arrhythmias. Heart **91**, 1352-1358. (10.1136/hrt.2004.046334) 16162633 PMC1769155

[15] Cubeddu L. 2016 Drug-induced inhibition and trafficking disruption of ion channels: pathogenesis of QT abnormalities and drug-induced fatal arrhythmias. Curr. Cardiol. Rev. **12**, 141-154. (10.2174/1573403X12666160301120217) 26926294 PMC4861943

[16] Bianchi L, Shen Z, Dennis AT, Priori SG, Napolitano C, Ronchetti E, Bryskin R, Schwartz PJ, Brown AM. 1999 Cellular dysfunction of LQT5-minK mutants: abnormalities of I_Ks_, I_Kr_ and trafficking in long QT syndrome. Hum. Mol. Genet. **8**, 1499-1507. (10.1093/hmg/8.8.1499) 10400998

[17] Zhang H, Hancox JC. 2004 In silico study of action potential and QT interval shortening due to loss of inactivation of the cardiac rapid delayed rectifier potassium current. Biochem. Biophys. Res. Commun. **322**, 693-699. (10.1016/j.bbrc.2004.07.176) 15325285

[18] Schwartz PJ et al. 2001 Genotype-phenotype correlation in the long-QT syndrome: gene-specific triggers for life-threatening arrhythmias. Circulation **103**, 89-95. (10.1161/01.CIR.103.1.89) 11136691

[19] Schwartz PJ, Ackerman MJ. 2013 The long QT syndrome: a transatlantic clinical approach to diagnosis and therapy. Eur. Heart J. **34**, 3109-3116. (10.1093/eurheartj/eht089) 23509228

[20] Priori SG et al. 2003 Risk stratification in the long-QT syndrome. New Engl. J. Med. **348**, 1866-1874. (10.1056/NEJMoa022147) 12736279

[21] Mehta A et al. 2018 Identification of a targeted and testable antiarrhythmic therapy for long-QT syndrome type 2 using a patient-specific cellular model. Eur. Heart J. **39**, 1446-1455. (10.1093/eurheartj/ehx394) 29020304

[22] Bellin M et al. 2013 Isogenic human pluripotent stem cell pairs reveal the role of a KCNH2 mutation in long-QT syndrome. EMBO J. **32**, 3161-3175. (10.1038/emboj.2013.240) 24213244 PMC3981141

[23] Moss AJ et al. 2007 Clinical aspects of type-1 long-QT syndrome by location, coding type, and biophysical function of mutations involving the KCNQ1 gene. Circulation **115**, 2481-2489. (10.1161/CIRCULATIONAHA.106.665406) 17470695 PMC3332528

[24] Anderson CL, Kuzmicki CE, Childs RR, Hintz CJ, Delisle BP, January CT. 2014 Large-scale mutational analysis of Kv11.1 reveals molecular insights into type 2 long QT syndrome. Nat. Commun. **5**, 5535. (10.1038/ncomms6535) 25417810 PMC4243539

[25] Kekenes-Huskey PM, Burgess DE, Sun B, Bartos DC, Rozmus ER, Anderson CL, January CT, Eckhardt LL, Delisle BP. 2022 Mutation-specific differences in Kv7.1 (KCNQ1) and Kv11.1 (KCNH2) channel dysfunction and long QT syndrome phenotypes. Int. J. Mol. Sci. **23**, 7389. (10.3390/ijms23137389) 35806392 PMC9266926

[26] Keating MT, Sanguinetti MC. 2001 Molecular and cellular mechanisms of cardiac arrhythmias. Cell **104**, 569-580. (10.1016/S0092-8674(01)00243-4) 11239413

[27] Curran ME, Splawski I, Timothy KW, Vincen GM, Green ED, Keating MT. 1995 A molecular basis for cardiac arrhythmia: HERG mutations cause long QT syndrome. Cell **80**, 795-803. (10.1016/0092-8674(95)90358-5) 7889573

[28] Lamothe SM, Guo J, Li W, Yang T, Zhang S. 2016 The human ether-a-go-go-related gene (hERG) potassium channel represents an unusual target for protease-mediated damage. J. Biol. Chem. **291**, 20 387-20 401. (10.1074/jbc.M116.743138) PMC5034037 27502273

[29] Yoshinaga M, Kucho Y, Sarantuya J, Ninomiya Y, Horigome H, Ushinohama H, Shimizu W, Horie M. 2014 Genetic characteristics of children and adolescents with long-QT syndrome diagnosed by school-based electrocardiographic screening programs. Circul. Arrhyth. Electrophysiol. **7**, 107-112. (10.1161/CIRCEP.113.000426) 24363352

[30] Al-Moubarak E, Zhang Y, Dempsey CE, Zhang H, Harmer SC, Hancox JC. 2020 Serine mutation of a conserved threonine in the hERG K^+^ channel S6-pore region leads to loss-of-function through trafficking impairment. Biochem. Biophys. Res. Commun. **526**, 1085-1091. (10.1016/j.bbrc.2020.04.003) 32321643 PMC7237882

[31] Adeniran I, McPate MJ, Witchel HJ, Hancox JC, Zhang H. 2011 Increased vulnerability of human ventricle to re-entrant excitation in hERG-linked variant 1 short QT syndrome. PLoS Comput. Biol. **7**, e1002313. (10.1371/journal.pcbi.1002313) 22194679 PMC3240585

[32] Whittaker DG, Ni H, Benson AP, Hancox JC, Zhang H. 2017 Computational analysis of the mode of action of disopyramide and quinidine on hERG-linked short QT syndrome in human ventricles. Front. Physiol. **8**, 759. (10.3389/fphys.2017.00759) 29085299 PMC5649182

[33] ten Tusscher KH, Noble D, Noble P-J, Panfilov AV. 2004 A model for human ventricular tissue. Am. J. Physiol. Heart Circul. Physiol. **286**, H1573-H1589. (10.1152/ajpheart.00794.2003) 14656705

[34] ten Tusscher KH, Panfilov AV. 2006 Cell model for efficient simulation of wave propagation in human ventricular tissue under normal and pathological conditions. Phys. Med. Biol. **51**, 6141. (10.1088/0031-9155/51/23/014) 17110776

[35] Ten Tusscher KH et al. 2009 Organization of ventricular fibrillation in the human heart: experiments and models. Exp. Physiol. **94**, 553-562. (10.1113/expphysiol.2008.044065) 19168541

[36] Broyden CG. 1970 The convergence of a class of double-rank minimization algorithms 1. General considerations. IMA J. Appl. Math. **6**, 76-90. (10.1093/imamat/6.1.76)

[37] O'Hara T, Virág L, Varró A, Rudy Y. 2011 Simulation of the undiseased human cardiac ventricular action potential: model formulation and experimental validation. PLoS Comput. Biol. **7**, e1002061. (10.1371/journal.pcbi.1002061) 21637795 PMC3102752

[38] Katz AM. 2010 Physiology of the heart. Philadelphia, PA: Lippincott Williams & Wilkins.

[39] Antzelevitch C. 2010 M cells in the human heart. Circ. Res. **106**, 815-817. (10.1161/CIRCRESAHA.109.216226) 20299671 PMC2859894

[40] Glukhov AV, Fedorov VV, Lou Q, Ravikumar VK, Kalish PW, Schuessler RB, Moazami N, Efimov IR. 2010 Transmural dispersion of repolarization in failing and nonfailing human ventricle. Circ. Res. **106**, 981-991. (10.1161/CIRCRESAHA.109.204891) 20093630 PMC2842469

[41] Wang W, Zhang S, Ni H, Garratt CJ, Boyett MR, Hancox JC, Zhang H. 2018 Mechanistic insight into spontaneous transition from cellular alternans to arrhythmia—a simulation study. PLoS Comput. Biol. **14**, e1006594. (10.1371/journal.pcbi.1006594) 30500818 PMC6291170

[42] You T, Luo C, Zhang K, Zhang H. 2021 Electrophysiological mechanisms underlying T-wave alternans and their role in arrhythmogenesis. Front. Physiol. **12**, 614946. (10.3389/fphys.2021.614946) 33746768 PMC7969788

[43] Adeniran I, El Harchi A, Hancox JC, Zhang H. 2012 Proarrhythmia in *KCNJ2*-linked short QT syndrome: insights from modelling. Cardiovasc. Res. **94**, 66-76. (10.1093/cvr/cvs082) 22308236

[44] Zhang H, Kharche S, Holden AV, Hancox JC. 2008 Repolarisation and vulnerability to re-entry in the human heart with short QT syndrome arising from KCNQ1 mutation—a simulation study. Prog. Biophys. Mol. Biol. **96**, 112-131. (10.1016/j.pbiomolbio.2007.07.020) 17905416

[45] Quan W, Rudy Y. 1990 Unidirectional block and reentry of cardiac excitation: a model study. Circ. Res. **66**, 367-382. (10.1161/01.RES.66.2.367) 2297808

[46] Zhang H, Garrat C, Holden AV. 2003 Onset and termination of reentrant excitation in homogeneous human virtual atrial tissue. Int. J. Bifurcation Chaos **13**, 3631-3643. (10.1142/S0218127403008867)

[47] Clayton R et al. 2011 Models of cardiac tissue electrophysiology: progress, challenges and open questions. Prog. Biophys. Mol. Biol. **104**, 22-48. (10.1016/j.pbiomolbio.2010.05.008) 20553746

[48] Ten Tusscher KH, Panfilov AV. 2006 Alternans and spiral breakup in a human ventricular tissue model. Am. J. Physiol. Heart Circul. Physiol. **291**, H1088-H1100. (10.1152/ajpheart.00109.2006) 16565318

[49] Keller DU, Jarrousse O, Fritz T, Ley S, Dossel O, Seemann G. 2011 Impact of physiological ventricular deformation on the morphology of the T-wave: a hybrid, static-dynamic approach. IEEE Trans. Biomed. Eng. **58**, 2109-2119. (10.1109/TBME.2011.2147785) 21536516

[50] Alday EAP, Ni H, Zhang C, Colman MA, Gan Z, Zhang H. 2016 Comparison of electric- and magnetic-cardiograms produced by myocardial ischemia in models of the human ventricle and torso. PLoS ONE **11**, e0160999. (10.1371/journal.pone.0160999) 27556808 PMC4996509

[51] Biktashev V, Holden A. 1998 Reentrant waves and their elimination in a model of mammalian ventricular tissue. Chaos Interdiscip. J. Nonlinear Sci. **8**, 48-56. (10.1063/1.166307) 12779709

[52] Kharche SR, Stary T, Colman MA, Biktasheva IV, Workman AJ, Rankin AC, Holden AV, Zhang H. 2014 Effects of human atrial ionic remodelling by β-blocker therapy on mechanisms of atrial fibrillation: a computer simulation. Europace **16**, 1524-1533. (10.1093/europace/euu084) 25085203 PMC4640177

[53] Keller D, Kalayciyan R, Dössel O, Seemann G. 2009 Fast creation of endocardial stimulation profiles for the realistic simulation of body surface ECGs. In World Congress on Medical Physics and Biomedical Engineering (eds O Dössel, WC Schlegel), pp. 145-148. Berlin, Germany: Springer. (10.1007/978-3-642-03882-2_37)

[54] Fernandes GC, Fernandes A, Cardoso R, Nasi G, Rivera M, Mitrani RD, Goldberger JJ. 2018 Ablation strategies for the management of symptomatic Brugada syndrome: a systematic review. Heart Rhythm **15**, 1140-1147. (10.1016/j.hrthm.2018.03.019) 29572085

[55] Wacker S, Noskov SY, Perissinotti LL. 2017 Computational models for understanding of structure, function and pharmacology of the cardiac potassium channel Kv11.1 (hERG). Curr. Top. Med. Chem. **17**, 2681-2702. (10.2174/1568026617666170414143430) 28413954

[56] Skibsbye L, Ravens U. 2016 Mechanism of proarrhythmic effects of potassium channel blockers. Card. Electrophysiol. Clin. **8**, 395-410. (10.1016/j.ccep.2016.02.004) 27261830

[57] Du D, Yang H, Norring SA, Bennett ES. 2011 Multi-scale modeling of glycosylation modulation dynamics in cardiac electrical signaling. In 2011 Annual Int. Conf. of the IEEE Engineering in Medicine and Biology Society, Boston, MA, USA, 30 August–3 September 2011. (10.1109/IEMBS.2011.6089907) 22254261

[58] Clancy CE, Rudy Y. 2001 Cellular consequences of HERG mutations in the long QT syndrome: precursors to sudden cardiac death. Cardiovasc. Res. **50**, 301-313. (10.1016/S0008-6363(00)00293-5) 11334834

[59] Clayton RH, Bailey A, Biktashev VN, Holden AV. 2001 Re-entrant cardiac arrhythmias in computational models of long QT myocardium. J. Theor. Biol. **208**, 215-225. (10.1006/jtbi.2000.2212) 11162065

[60] Keller DU, Seemann G, Weiss DL, Farina D, Zehelein J, Dossel O. 2007 Computer based modeling of the congenital long-QT 2 syndrome in the visible man torso: from genes to ECG. In 2007 29th Annual Int. Conf. of the IEEE Engineering in Medicine and Biology Society, Lyon, France, 22–26 August 2007. (10.1109/IEMBS.2007.4352563) 18002229

[61] Zheng Z, Song Y, Lian J. 2022 What is the potential for lumacaftor as a chemical chaperone in promoting hERG trafficking? Front. Cardiovasc. Med. **9**, 801927. (10.3389/fcvm.2022.801927) 35282377 PMC8913575

[62] Hancox JC, Stuart AG, Harmer SC. 2020 Functional evaluation of gene mutations in long QT syndrome: strength of evidence from in vitro assays for deciphering variants of uncertain significance. J. Congenit. Cardiol. **4**, 6. (10.1186/s40949-020-00037-9)

[63] Ni H, Zhang H, Grandi E, Narayan SM, Giles WR. 2019 Transient outward K^+^ current can strongly modulate action potential duration and initiate alternans in the human atrium. Am. J. Physiol. Heart Circul. Physiol. **316**, H527-H542. (10.1152/ajpheart.00251.2018) PMC6415821 30576220

[64] El-Sherif N, Turitto G, Boutjdir M. 2018 Acquired long QT syndrome and torsade de pointes. Pacing Clin. Electrophysiol. **41**, 414-421. (10.1111/pace.13296) 29405316

[65] Pang JK, Chia S, Zhang J, Szyniarowski P, Stewart C, Yang H, Chan W-K, Ng SY, Soh B-S. 2022 Characterizing arrhythmia using machine learning analysis of Ca^2+^ cycling in human cardiomyocytes. Stem Cell Rep. **17**, 1810-1823. (10.1016/j.stemcr.2022.06.005) PMC9391413 35839773

[66] Singstad B-J, Dalen BS, Sihra S, Forsch N, Wall S. 2022 Identifying ionic channel block in a virtual cardiomyocyte population using machine learning classifiers. In Computational physiology (ed. KJ McCabe), pp. 91-109. Cham, Switzerland: Springer. (10.1007/978-3-031-05164-7_8)

[67] Draelos RL, Ezekian JE, Zhuang F, Moya-Mendez ME, Zhang Z, Rosamilia MB, Manivannan PK, Henao R, Landstrom AP. 2022 GENESIS: gene-specific machine learning models for variants of uncertain significance found in catecholaminergic polymorphic ventricular tachycardia and long QT syndrome-associated genes. Circul. Arrhyth. Electrophysiol. **15**, e010326. (10.1161/CIRCEP.121.010326) PMC9018586 35357185

[68] Jiang C et al. 2022 A calibrated functional patch-clamp assay to enhance clinical variant interpretation in KCNH2-related long QT syndrome. Am. J. Hum. Genet. **109**, 1199-1207. (10.1016/j.ajhg.2022.05.002) 35688147 PMC9300752

[69] Haraguchi Y, Ohtsuki A, Oka T, Shimizu T. 2015 Electrophysiological analysis of mammalian cells expressing hERG using automated 384-well-patch-clamp. BMC Pharmacol. Toxicol. **16**, 39. (10.1186/s40360-015-0042-9) 26671227 PMC4681162

[70] Hu W, Zhang W, Zhang K, Al-Moubarak E, Zhang Y, Harmer SC, Hancox JC, Zhang H. 2023 Evaluating pro-arrhythmogenic effects of the T634S-hERG mutation: insights from a simulation study. *Figshare*. (10.6084/m9.figshare.c.6935455) PMC10722218 38106919

